# Characterization of Ras Y4H mutants in *Drosophila*

**DOI:** 10.17912/micropub.biology.001068

**Published:** 2024-02-29

**Authors:** Prashath Karunaraj, Chalita Washington, Max Luf, Yesenia Martino-Cortez, Cathie M Pfleger

**Affiliations:** 1 Oncological Sciences, Icahn School of Medicine at Mount Sinai; 2 The Tisch Cancer Institute; 3 The Graduate School of Biomedical Sciences

## Abstract

Ras signaling plays a highly conserved role from flies to mammals in establishing proper development, and its dysregulation can lead to cancer. In *Drosophila* , we demonstrated that Ras Tyrosine 4 (Y4) was required for inhibitory ubiquitination by Rabex-5. In humans, rare histidine substitution mutations at Y4 are found in HRas in cerebellar glioblastomas (cGBMs). We report here that analogous Y4H mutations in *Drosophila* Ras make it less sensitive to Rabex-5-mediated ubiquitination in cells and show increased frequency of vein phenotypes per wing compared to wild-type Ras, which would be consistent with Ras gain-of-function and with their appearance in human cGBMs.

**Figure 1. Ras Y4H is insensitive to Rabex-5-mediated ubiquitination and shows increased frequency of wing vein phenotypes compared to Ras WT in vivo .**
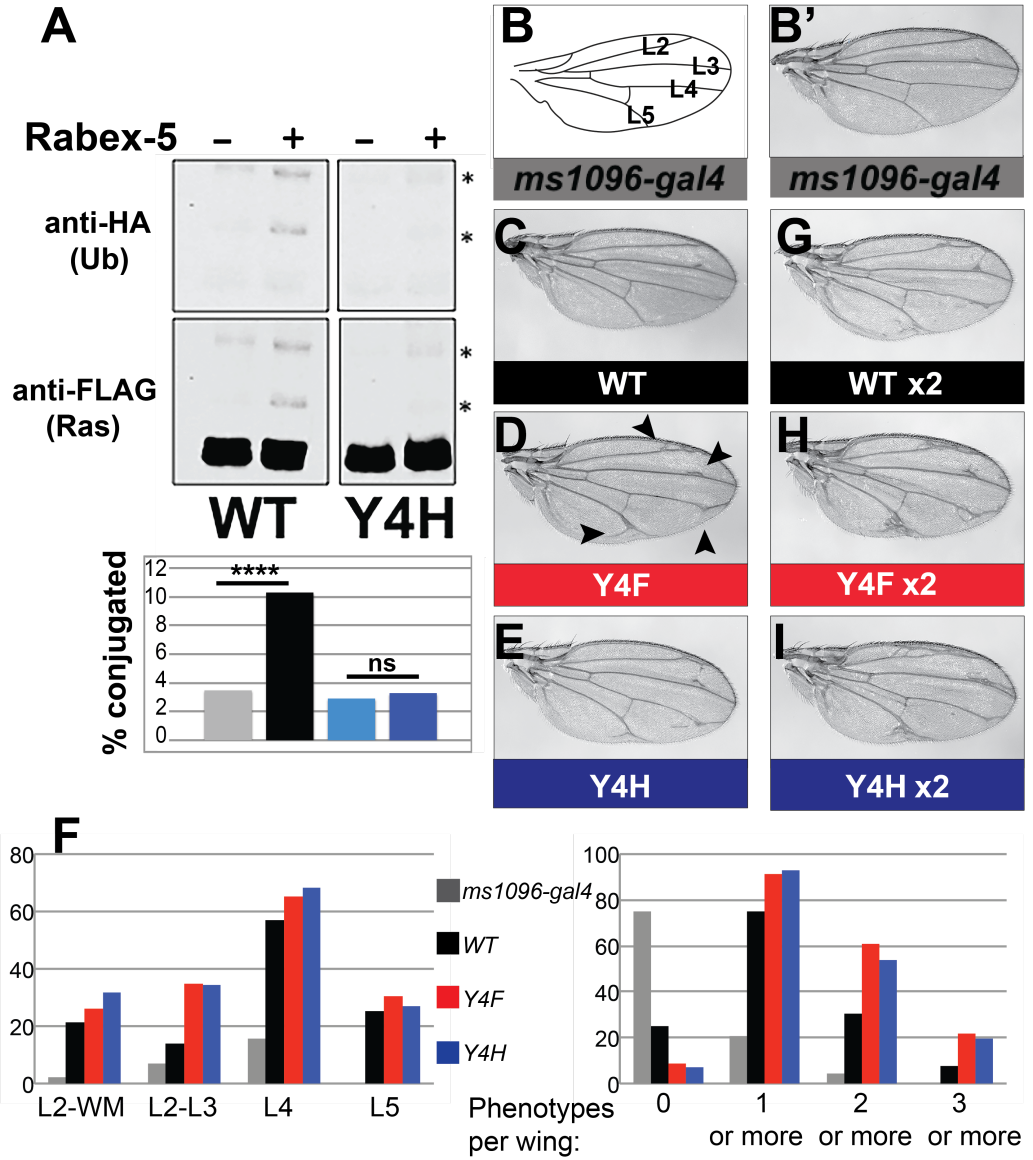
(A) S2 cells were transfected with FLAG-HIS6 tagged Ras ^WT^ or Ras ^Y4H^ and HA-Ubiquitin. Western blots were stained with anti-HA (to label ubiquitin conjugates, upper panels) or anti-FLAG (to label Ras and Ras ubiquitin conjugates, lower panels). Ras ^WT^ showed increased ubiquitination when co-transfected with Rabex-5 but Ras ^Y4H^ did not. Mono- and di-ubiquitin conjugates in both sets of gels are labeled to the right with an asterisk. Quantification of anti-FLAG gels shows percent of Ras in ubiquitin conjugates in graph under the Western panels. **** indicates p<0.0001 and ns indicates not significant in Fisher’s exact test and Chi-square tests as calculated in GraphPad Prism. (B) Schematic of a wing labeling L2, L3, L4, and L5 longitudinal veins in a wing with normal morphology. (B’) Control *ms1096-gal4* wing. (C-E) *ms1096-gal4* driving expression of one copy of (C) *UAS Ras ^WT^* , (D) *UAS Ras ^Y4F^* , and (E) *UAS Ras ^Y4H^* . Under conditions where Ras ^WT^ expression causes very little phenotype, Ras ^Y4F^ and Ras ^Y4H^ expression both cause increased wing vein abnormalities associated with Ras gain-of-function. Abnormalities are most notable between L2 and the wing margin, between L2 and L3, and where L4 and L5 meet with wing margin (examples of these specific phenotypes are indicated by arrowheads in 1D). (F) We scored all wings expressing one copy of the indicated transgene (or *ms1096-gal4* control) for the presence of phenotypes between L2 and the wing margin (L2-WM), between L2 and L3 (L2-L3), where L4 meets the wing margin (L4), or where L5 meets the wing margin (L5). The left graph indicates the percentage of wings in a typical trial with each of these phenotypes per genotype. In this trial, there is a clear difference between genotypes for L2-L3, but this was not true in every trial; we saw variability between trials for specific phenotypes. We also quantified the number of phenotypes per wing (no phenotypes, 1 or more, 2 or more, or 3 or more phenotypes. The right graph indicates the percentage of wings in each of these categories per genotype. Despite the variability at specific vein positions, reproducibly all three trials scored were highly reproducible for the increased frequency of phenotypes. We saw statistically significant differences between Ras ^WT^ and Ras ^Y4H^ using Chi-square and Fisher’s exact tests in Graphpad Prism when comparing categories of no phenotypes versus 1 or more phenotypes or 0-1 phenotype versus 2 or more phenotypes (see Statistical analysis section in Methods for p values and details). Although we reproducibly saw differences between categories of less than 3 phenotypes versus 3 or more phenotypes per wing, this was not always statistically significant possibly due to the lower number of wings with 3 or more phenotypes. (G-I) *ms1096-gal4* wings homozygous for (G) *UAS Ras ^WT^* , (H) *UAS Ras ^Y4F^* , and (I) *UAS Ras ^Y4H^* . Male wings are shown in B-H. Female wings of the same genotypes show weaker phenotypes but the same phenomena.

## Description

Ras (called Ras85D and Ras1 in *Drosophila* , here referred to as “Ras”) is an important regulator of development and is dysregulated in Rasopathies and cancer. Ras is represented by one gene in *Drosophila* and by HRas, NRas, and KRas in mammals. *Drosophila* Ras and HRas and NRas are negatively regulated by inhibitory ubiquitination [Jura et al., 2006; Yan et al., 2009] by the E3 Rabex-5 [Yan et al. 2010, Xu et al 2010]. We previously showed that Rabex-5 requires Tyrosine 4 (Y4) to promote Ras ubiquitination in *Drosophila* [Washington et al., 2020]; Y4F mutations cause Ras to evade ubiquitination by Rabex-5 *in vitro* and behave as gain-of-function Ras mutations *in vivo* [Washington et al. 2020].

Curiously, Y4 mutations in HRas, HRas ^Y4H^ , have been reported in cerebellar glioblastoma multiforme (cGBM) [Milinkovic et al., 2014], a rare form of glioblastoma multiforme (GBM). Because our previous work demonstrated the importance of Y4 for Ras recognition by Rabex-5 [Washington et al., 2020], we speculated that these Y4H mutations might act like gain-of-function mutations by evading inhibition by Rabex-5 to aid in cerebellar glioblastoma growth. We engineered Y4H mutations in *Drosophila* Ras, Ras ^Y4H^ , in pIE ^1-4^ vectors for tissue culture work and pUASTattb for generating transgenic lines with site-specific integration. Ras ^Y4H^ was tagged N-terminally with FLAG-HIS6 tags used for wild type Ras, Ras ^WT^ , and Ras ^Y4F^ as in our previous work [Washington et al., 2020]. Transgenic lines used insertion at the attp40 site, the same genomic site as previously generated lines *UAS Ras ^WT^* and *UAS Ras ^Y4F^* , so any difference in phenotype would be due to the specific Y4 mutation and not position insertion effects.

We isolated Ras ubiquitin conjugates from cells using previous protocols [Yan et al., 2009; Yan et al., 2010; Washington et al., 2020]. S2 cells were transfected with Ras constructs and HA-UB plasmid with vector control or Rabex-5, and Ras was pulled down on nickel beads and then analyzed by Western. Co-transfection of S2 cells with Rabex-5 increased the ubiquitination of Ras ^WT^ above baseline to a far greater extent than seen with Ras ^Y4H^ ( Fig. 1A ). *In vivo* , expressing Ras ^WT^ using *ms1096-gal4* results in wings with some extra wing vein material ( Fig. 1C ) compared to the normal vein pattern of control wings ( Fig. 1B ’, schematic of wing veins shown in Fig. 1B ) most notably disrupted or ectopic vein material where L4 meets the wing margin but also some frequency of disrupted or ectopic vein material between L2 and the wing margin, between L2 and L3, and disrupted or ectopic vein material where L5 meets the wing margin. As we reported previously [Washington et al., 2020], expressing Ras ^Y4F^ causes increased abnormalities compared to Ras ^WT^ ( Fig. 1D, quantified in Figure 1F). Expressing Ras ^Y4H^ resembles Ras ^Y4F^ and shows increased abnormalities compared to Ras ^WT^ ( Fig. 1E, quantified in Figure 1F). Because these wing phenotypes are variable, to establish the increase in wing vein abnormalities, three different lab members scored male wings expressing one copy of each transgene with *ms1096-gal4* compared to control *ms1096-gal4* wings for the presence or absence of a phenotype at four sites: between L2 and the wing margin, between L2 and L3, where L4 meets the wing margin, and where L5 meets the wing margin ( Fig. 1F left graph, phenotypes evident in wings shown in Fig . 1D-1E, 1G-1I and noted by arrowheads in 1D). We see some background of phenotypes in control wings, and the incidence of phenotypes increases upon Ras ^WT^ expression as is seen throughout the literature. We saw variability in the incidence of specific phenotypes between trials, so we cannot conclude that Ras ^Y4F^ and Ras ^Y4H^ cause greater frequency of the L2-L3 phenotype as shown in the trial depicted in Fig. 1F . We did not see reproducible differences in severity between Ras ^Y4F^ and Ras ^Y4H^ . However, reproducibly in the three data sets scored, the number of phenotypes per wing statistically significantly increases across the population for Ras ^Y4H^ compared to Ras ^WT^ ( Fig. 1F, right graph ). Taken together, the failure of Rabex-5 to increase Ras ^Y4H^ ubiquitination *in vitro* to the extent it did for Ras ^WT^ ( Fig. 1A ) and the increased frequency of phenotypes per wing of Ras ^Y4H^ *in vivo* compared to Ras ^WT^ ( Fig. 1F ) are consistent with a model that Ras Y4H acts as a gain-of-function Ras mutation by evading Rabex-5 inhibition. Increasing the gene dosage by generating flies homozygous for the Ras transgenes showed stronger wing vein phenotypes; subjective observations by independent observers scored these phenotypes as stronger across the population for Ras ^Y4F^ ( Fig. 1H ) and Ras ^Y4H^ ( Fig. 1I ) compared to Ras ^WT^ ( Fig. 1G ), however relative severity of this was not quantified due to the more subjective nature of such scoring. Future work will define corresponding increases in molecular readouts of Ras activity, such as an increase in the relative proportion of dpERK, and will explore the conservation of this result to elucidate if HRas ^Y4H^ variants identified in cGBMs underlie the etiology of cGBMs by also evading Ras ubiquitination to act as Ras gain-of-function mutations.

## Methods

**Reproducibility:** The reported work represents reproducible experiments that reflect a minimum of three well-controlled, independent trials.

**Tissue Culture.** S2 cells were cultured using standard methods at 25°C in Schneider’s *Drosophila* Medium (1X) (Gibco, 21720-024). Cells were transfected using Effectene Transfection Reagent (Qiagen, Cat # 301427) according to manufacturer instructions with plasmids *pUAST-HA-Ub, Act-gal4, UAS myc Rabex-5, pIE ^1-4^ FLAG-His6 Ras ^WT^ , pIE ^1-4^ FLAG-His6 Ras ^Y4F^ ,* and *pIE ^1-4^ FLAG-His6 Ras ^Y4H^* as indicated and harvested after 48-72 hours. The amounts of *pUAST-HA-Ub and Act-gal4* (to direct ubiquitin over-expression) plasmids were kept constant across experiments. Constructs are summarized in Table 1, and protein sequences of Ras constructs are detailed in Table 2.

**Schneider S2 cell extract preparation and Nickel pull down of tagged Ras from Schneider S2 cells (for Western analysis)** : Schneider S2 cell extracts were prepared and used to isolate tagged Ras in Figure 1A as described previously [Washington et al., Yan et al. 2009; Yan et al 2010]. Extracts were prepared from harvested cells or from frozen cell pellets in lysis buffer, 1X NP40 buffer (50 mM HEPES pH 7.4, 1% NP40, 1mM EDTA, 150 mM NaCl) *,* 8M Urea, 5 mM beta-mercaptoethanol, 10 mM imidazole supplemented with Roche protease inhibitor cocktail and 1mM each of PMSF and Sodium Orthovanadate) and used in pull down assays described above or analyzed by Western. Ni-NTA agarose beads (Qiagen) were equilibrated per manufacturer’s instructions, washed twice with binding buffer, pH 8.0 (1X PBS, 8 M Urea, 0.5% NP40, 10 mM Imidazole, 360 mM NaCl), then incubated with binding buffer, pH 8.0 supplemented with 1g/ml BSA. Lysates were incubated with beads at 4°C for 2 hours. Beads were transferred to mini-columns on a vacuum assembly and washed 3X with wash buffer pH 6.3 (1X PBS, 8 M Urea, 0.5% NP40, 30 mM Imidazole, 360 mM NaCl). Proteins were eluted from the beads with elution buffer pH 6.3 (1X PBS, 8 M Urea, 0.5% NP40, 500 mM Imidazole, 360 mM NaCl), boiled in 1X loading buffer for 10 minutes, then stored at -20°C or run immediately on a gel.

**Western Blot analysis** . Western blots used Immobilon-FL Transfer Membrane (Millipore, Cat # IPFL00010) and were visualized using the Li-Cor Odyssey System. Primary antibodies were anti-FLAG (rabbit, Sigma Catalog # F7425-.2MG, 1:1000), anti-HA (mouse, Roche Catalog # 12CA5, 1:1000), secondary antibodies were Alexa Fluor goat anti-mouse 488 (Invitrogen, Catalog # A11001, 1:10,000), Alexa Fluor goat anti-rabbit 680 (Invitrogen, Catalog # A21076, 1:10,000), Alexa Fluor goat anti-mouse 680 (Invitrogen, Catalog # A21057, 1:10,000), DyLight goat anti-mouse 800 (Thermo Scientific, Catalog # 35521, 1:10,000), and DyLight goat anti-rabbit 800 (Thermo Scientific, Catalog # 35571, 1:10,000). Raw tiff files from the Li-Cor Odyssey were split from full color into specific channels corresponding to single antibodies that were then converted to grayscale using Adobe Photoshop. Images were cropped to fit figure panels from different sections of the same gel (to crop out unrelated experiments); adjustments to brightness and contrast were applied uniformly to the entire images not to portions of an image before cropping. Image was converted to 500 dpi within Photoshop to adhere to journal image requirements.

**Quantification of ubiquitination** . Quantification of percent ubiquitination utilized gels in which signals were not over-saturated. To quantify percent ubiquitination, gel lanes were analyzed using Image J; we summed the signal of unconjugated and ubiquitinated Ras bands in each lane to define total Ras signal in that lane and then calculated the proportion of each band per total to indicate percent of Ras conjugated to ubiquitin out of total Ras using a method used previously [Washington et al., 2020]. In this manner, we could make comparisons of percent conjugation to ubiquitin in one lane to the percent conjugation to ubiquitin in another lane from different lanes on the same gel within the same experiment.

**Statistical analysis comparing ubiquitin conjugates.** Statistical analysis comparing the percent of conjugated and unconjugated Ras shown in Figure 1A (as described above) used Fisher’s exact test and Chi-square tests in Graphpad Prism.

**Statistical analysis comparing the incidence of wing vein phenotypes.** We compared categories of no phenotypes versus 1 or more phenotypes; 0 and 1 phenotype versus 2 or more phenotypes; or less than 3 phenotypes versus 3 or more phenotypes per wing in control *ms1096-gal4* wings compared to *ms1096-gal4* driving Ras transgenes or of *ms1096-gal4* driving Ras ^WT^ compared to *ms1096-gal4* driving Ras ^Y4H^ with both Chi-square and Fisher’s exact tests in Graphpad Prism. We saw statistically significant differences between all three transgenes and control *ms1096-gal4* wings when comparing no phenotypes versus 1 or more (p<0.0001 for all cases), when comparing 0-1 phenotype versus 2 or more phenotypes (p=0.0008 in Chi-square and p=0.0005 in Fisher’s exact test for control *ms1096-gal4* versus Ras ^WT^ and p<0.0001 for control *ms1096-gal4* versus each Y4 mutant). We saw no difference between control *ms1096-gal4* and Ras ^WT^ when comparing less than 3 phenotypes versus 3 or more phenotypes (p=0.0609 in Chi-square and p=0.0873 in Fisher’s exact test), but statistically significant difference when comparing control *ms1096-gal4* to Ras ^Y4F^ (p=0.0013 in Chi-square and p=0.0035 in Fisher’s exact test) or Ras ^Y4H^ (p=0.0021 in Chi-square and p=0.0020 in Fisher’s exact test). We saw statistically significant differences between Ras ^WT^ and Ras ^Y4H^ using Chi-square and Fisher’s exact tests in Graphpad Prism when comparing categories of no phenotypes versus 1 or more phenotypes (p=0.0175 in Chi-square test and p=0.0260 in Fisher’s exact test) or 0-1 phenotype versus 2 or more phenotypes (p=0.029 in Chi-square test and p=0.0175 in Fisher’s exact test). Although we reproducibly saw differences between categories of less than 3 phenotypes versus 3 or more phenotypes per wing, this was not always statistically significant possibly due to the lower number of wings with 3 or more phenotypes.

***Drosophila* experiments** . UAS Ras ^Y4H^ was cloned into pUAST-attB with the FLAG and His6 sequences of MDYKDDDDKRGSHHHHHHALE preceding the Ras coding sequence as we did previously with *UAS FLAG-His6-Ras ^WT^* and *UAS Flag-His6-Ras ^Y4F^* . The plasmid was sent to BestGene for injection and generation of transgenic lines at the attp40 locus. Lines were balanced over CyO or SM6-TM6B balancers and then maintained as true-breeding homozygous stocks. Genomic DNA was sequenced to confirm each insert. Importantly, UAS Ras ^Y4H^ differs in sequence from UAS Ras ^Y4F^ and UAS Ras ^WT^ only at Y4. Crosses were performed at the indicated temperatures on standard *Drosophila* medium. Raw wing images were converted to grayscale using Adobe Photoshop. Brightness and contrast of wing images were adjusted using Adobe Photoshop to maximize clarity; adjustments were applied to the entire images. Genotypes are summarized below, and identifiers are annotated in Table 1. Protein sequences for Ras transgenes are detailed in Table 2.

**Genotypes of fly images**

*ms1096-gal4* (Fig.1B’, left-most/gray bar in graphs in F)

*ms1096-gal4; UAS Ras ^WT^ /+* ( Fig. 1C, 2 ^nd^ /black bar in graphs in F)

*ms1096-gal4; UAS Ras ^Y4F^ /+* ( Fig. 1D, 3 ^rd^ /red bar in graphs in F)

*ms1096-gal4; UAS Ras ^Y4H^ /+* ( Fig. 1E, right -most/blue bar in graphs in F)

*ms1096-gal4; UAS Ras ^WT^* ( Fig. 1G )

*ms1096-gal4; UAS Ras ^Y4F^* ( Fig. 1H )

*ms1096-gal4; UAS Ras ^Y4H^* ( Fig. 1I )

## Reagents

**Table 1: Table of reagents used in the manuscript with corresponding identifiers.**

**Table:** 

**REAGENT or RESOURCE**	**SOURCE**	**IDENTIFIER**
**Primary Antibodies**		
Rabbit polyclonal anti-FLAG primary antibodies	Sigma	Catalog #: F7425-.2MG
Mouse monoclonal anti-HA primary antibodies	Roche	Catalog #: 12CA5
**Secondary antibodies**		
Goat anti-mouse IgG (H+L) Alexa Fluor 488	Molecular Probes – Invitrogen	Catalog #: A11001
Goat anti-mouse IgG (H+L) Alexa Fluor 680	Molecular Probes – Invitrogen	Catalog #: A21057
Goat anti-rabbit IgG (H+L) Alexa Fluor 680	Molecular Probes – Invitrogen	Catalog #: A21076
Goat anti-mouse IgG (H+L) DyLight 800 Conjugated secondary antibodies	Thermo Scientific	Catalog #: 35521
Goat anti-rabbit IgG (H+L) DyLight 800 conjugated secondary antibodies	Thermo Scientific	Catalog #: 35571
**Cell Culture and biochemistry reagents**		
Qiagen Ni-NTA Agarose	QIAGEN	Catalog # 30230
COMPLETE EDTA free protease inhibitor Cocktail	Roche	Catalog # 11873580001
Phosphosafe Extraction Buffer	Sigma Aldrich	Catalog # 71296-3
Phenylmethylsulfonyl Fluoride	Sigma	Catalog # S-6508
Schneider’s Drosophila Medium (1X)	GIBCO	21720-024
Effectene Transfection Reagent	QIAGEN	Catalog # 301427
**Drosophila Strains**		
* w ^1118^*	The fly community and Bloomington Drosophila Stock Center (BDSC)	BL-3605, BL-5905 and others RRID:BDSC_3605, RRID:BDSC_5905
*ms1096-gal4*	BDSC	BL-8696 RRID:BDSC_8696
* UAS FLAG-His6-Ras ^WT^*	Washington et al., 2020	
* UAS FLAG-His6-Ras ^Y4F^*	Washington et al., 2020	
* UAS FLAG-His6-Ras ^Y4H^*	This study	
**Experimental Models: Cell Lines**		
*Drosophila* S2 cells	A gift from the NYC fly community	
**Recombinant DNA**		
*pUAST-HA-Ub*	Yan et al. 2010	
*Act-gal4*	A gift from the Mlodzik lab, also used in Yan et al. 2010	
*UAS myc Rabex-5*	Yan et al. 2010	
* pIE ^1-4^ FLAG-His6 Ras WT *	Yan et al. 2010	
* pIE ^1-4^ FLAG-His6 Ras Y4F *	Washington et al., 2020	
* pIE ^1-4^ FLAG-His6 Ras Y4H *	This study	
**Software**		
Image J		https://imagej.nih.gov/ij/
Adobe Photoshop		https://www.adobe.com/products/photoshop.html
GraphPad Prism		https://www.graphpad.com/scientific-software/prism/
Microsoft Excel		https://www.microsoft.com/Microsoft/Excel/

**Table 2: Table of protein sequences for Ras constructs used *in vitro* and *in vivo* .**

**Table:** 

Construct (as listed in Table 1)	Protein sequence Mutations: **bold, underlined** Tags: FLAG= **DYKDDDDK** His6= **HHHHHH**
* UAS FLAG-His6-Ras ^WT^*	MDYKDDDDKRGSHHHHHHALEMTEYKLVVVGAGGVGKSALTIQLIQNHFVDEYDPTIEDSYRKQVVIDGETCLLDILDTAGQEEYSAMRDQYMRTGEGFLLVFAVNSAKSFEDIGTYREQIKRVKDAEEVPMVLVGNKCDLASWNVNNEQAREVAKQYGIPYIETSAKTRMGVDDAFYTLVREIRKDKDNKGRRGRKMNKPNRRFKCKML
* UAS FLAG-His6-Ras ^Y4F^*	MDYKDDDDKRGSHHHHHHALEMTE **F** KLVVVGAGGVGKSALTIQLIQNHFVDEYDPTIEDSYRKQVVIDGETCLLDILDTAGQEEYSAMRDQYMRTGEGFLLVFAVNSAKSFEDIGTYREQIKRVKDAEEVPMVLVGNKCDLASWNVNNEQAREVAKQYGIPYIETSAKTRMGVDDAFYTLVREIRKDKDNKGRRGRKMNKPNRRFKCKML
* UAS FLAG-His6-Ras ^Y4H^*	MDYKDDDDKRGSHHHHHHALEMTE **H** KLVVVGAGGVGKSALTIQLIQNHFVDEYDPTIEDSYRKQVVIDGETCLLDILDTAGQEEYSAMRDQYMRTGEGFLLVFAVNSAKSFEDIGTYREQIKRVKDAEEVPMVLVGNKCDLASWNVNNEQAREVAKQYGIPYIETSAKTRMGVDDAFYTLVREIRKDKDNKGRRGRKMNKPNRRFKCKML

## References

[R1] Jura N, Scotto-Lavino E, Sobczyk A, Bar-Sagi, D. 2006. Differential modification of Ras proteins by ubiquitination. Mol. Cell. 21: 679-687. PMID: 16507365. 10.1016/j.molcel.2006.02.011 16507365

[R2] Milinkovic VP, Skender Gazibara MK, Manojlovic Gacic EM, Gazibara TM, Tanic NT. 2014. The impact of TP53 and RAS mutations on cerebellar glioblastomas. Exp Mol Pathol. 97: 202-207. PMID: 25036404. 10.1016/j.yexmp.2014.07.009 25036404

[R3] Washington C, Gokhale RH, Martino-Cortez Y, Liu H-Y, Rosenberg AM, Shahar S, Pfleger CM. 2020. A conserved, N-terminal tyrosine signal directs Ras for inhibition by Rabex-5. PLoS Genet 16: e1008715. PMID: 32559233. 10.1371/journal.pgen.1008715 PMC7329146 32559233

[R4] Xu L, Lubkov V, Taylor LJ, Bar-Sagi D. 2010. Feedback regulation of Ras signaling by Rabex-5-mediated ubiquitination. Curr Biol. 20: 1372-1377. PMID: 20655225. 10.1016/j.cub.2010.06.051 PMC3436604 20655225

[R5] Yan H, Chin M-L, Horvath EA, Kane EA, Pfleger CM. 2009. Impairment of ubiquitylation by mutation in *Drosophila* E1 promotes both cell-autonomous and non-cell-autonomous Ras-ERK activation *in vivo* . J Cell Science; 122: 1461-1470. PMID: 19366732. 10.1242/jcs.042267 PMC2721006 19366732

[R6] Yan H, Jahanshahi M, Horvath EA, Liu H-Y, Pfleger CM. Rabex-5 ubiquitin ligase activity restricts Ras signaling to establish pathway homeostasis in vivo in Drosophila. Current Biology 2010; 20:1378-1382. PMID : 20655224. 10.1016/j.cub.2010.06.058 PMC2938185 20655224

